# Ischemic Postconditioning Decreases Cerebral Edema and Brain Blood Barrier Disruption Caused by Relief of Carotid Stenosis in a Rat Model of Cerebral Hypoperfusion

**DOI:** 10.1371/journal.pone.0057869

**Published:** 2013-02-28

**Authors:** Fuwei Yang, Xiaojie Zhang, Ying Sun, Boyu Wang, Chuibing Zhou, Yinan Luo, Pengfei Ge

**Affiliations:** 1 Department of Neurosurgery, First Bethune Hospital of Jilin University, Changchun, China; 2 Department of Neurosurgery, Changchun Children Hospital, Changchun, China; 3 Department of Neurosurgery, Second Hospital Affiliated to Harbin Medical University, Harbin, China; University of Pittsburgh, United States of America

## Abstract

**Background and Purpose:**

Complications due to brain edema and breakdown of blood brain barrier are an important factor affecting the treatment effects of patients with severe carotid stenosis. In this study, we investigated the protective effects of ischemic postconditioning on brain edema and disruption of blood brain barrier via establishing rat model of hypoperfusion due to severe carotid stenosis.

**Methods:**

Wistar rat model of hypoperfusion due to severe carotid stenosis was established by binding a stainless microtube to both carotid arteries. Ischemic postconditioning procedure consisted of three cycles of 30 seconds ischemia and 30 seconds reperfusion. Brain edema was evaluated by measuring cerebral water content, and blood brain barrier permeability was assayed by examining cerebral concentration of Evans' Blue (EB) and fluorescein sodium (NaF). ELISA was used to analyze the expression of MMP-9, claudin-5 and occludin. The activity and location of MMP-9 was analyzed by gelatin zymography and in situ zymography, respectively. The distribution of tight junction proteins claudin-5 and occludin was observed by immunohistochemistry.

**Results:**

The increased brain water content and cerebral concentration of EB and NaF were suppressed by administration of ischemic postconditioning prior to relief of carotid stenosis. Zymographic studies showed that MMP-9 was mainly located in the cortex and its activity was significantly improved by relief of carotid stenosis and, but the elevated MMP-9 activity was inhibited markedly by ischemic postconditioning. Immunohistochemistry revealed that ischemic postconditioning improved the discontinuous distribution of claudin-5 and occludin. ELISA detected that the expression of up-regulated MMP-9 and down-regulated claudin-5 and occludin caused by carotid relief were all attenuated by ischemic postconditioning.

**Conclusions:**

Ischemic postconditioning is an effective method to prevent brain edema and improve BBB permeability and could be used during relief of severe carotid stenosis.

## Introduction

Carotid artery stenosis remains a major public health issue in the developed countries [1]. Clinical study showed that severe carotid stenosis (>70%) would lead to symptoms related to transient ischemic attack and ischemic stroke [2], and even cognition dysfunction due to long-term cerebral hypoperfusion[3]. Data from animal experiment showed that 50% of the neurons in rat hippocampus CA1 region died at 30 days when severe bilateral carotid artery stenosis was produced [4]. Moreover, it is thought that the brain damage in chronic cerebral hypoperfusion rat models was due to the cerebral hypoperfusion at early stage [5]. Additionally, Wu et al reported that early revascularization within 7 days can be safely performed and was preferred over delaying operative treatment in the symptomatic carotid stenosis patients without evidence of intracerebral hemorrhage, carotid occlusion, or permanent neurologic deficits [6]. Thus, revascularization of stenotic artery at early stage and recovery of cerebral blood flow has become the treatment strategy for the patients with severe carotid stenosis [7], [8].

Despite clinical observations and animal experiments demonstrated that the damaged neurological function was improved when carotid stenosis was relieved [9], [10], postoperative “hyperfusion or reperfusion syndrome” consisting of headache, brain edema, and even intracranial hemorrhage, has been the main issues affecting the prognosis of these patients [11], [12]. It has been thought that the syndrome is associated with impaired cerebral auto-regulation of cerebral blood flow, but a recent study using perfusion MRI demonstrated a subgroup of patients who developed reperfusion syndrome with only moderate increases in CBF on transcranial Doppler (TCD) assessment [13]. Moreover, other clinical findings showed that blood brain barrier is involved this pathological process. Ivens S et al described a patient with cerebral hyperperfusion syndrome, whose imaging demonstrated early ipsilateral blood-brain barrier (BBB) breakdown with electroencephalographic evidence of cortical dysfunction preceding brain edema [14]. Similarly, Kokuzawa J et al found that pure vasogenic edema appeared following recovery of cerebral blood flow. These reports indicated that disruption of blood brain barrier was involved in the pathological course of cerebral hyperperfusion syndrome following recovery of cerebral blood flow [15]. Although studies have shown that brain edema and intracranial hemorrhage were related to disruption of BBB which is responsible for limiting and regulating the movement of plasma constituents into the brain parenchyma [16], it is needed to clarify whether relief of carotid artery stenosis at early stage would result in BBB disruption and elucidate its mechanism. Therefore, we investigated this issue in the present study by using a rat model of cerebral hypoperfusion.

Animal study has demonstrated that increased permeability of BBB was caused by aberrant higher activity and expression of matrix metalloproteinases such as MMP-9 and MMP-2 [17]–[19]. However, accumulating evidences revealed that MMP-9 (matrix metalloproteinase 9) played a crucial role in modulating the permeability of BBB under various pathological conditions such as head trauma, acute liver failure, focal or global ischemia and reperfusion [17]–[19]. Particularly, it was reported that MMP-9 activity elevated significantly in humans after stroke [20]. By contrast, inhibition of MMP-9 either by synthetic inhibitor or by specific monoclonal antibodies was proved to be an effective way to reverse BBB breakdown and attenuate brain extravasation and edema [21]. By now, evidences showed that MMP-9 modulating BBB permeability is via degradation of BBB constituents, such as tight junction proteins occludin and claudin-5, and basal lamina proteins fibronectin, laminin and heparan sulfate [22], [23]. Therefore, we focused on MMP-9, occludin and claudin-5 in this study to investigate the mechanism of BBB disruption caused by early relief of carotid stenosis.

Ischemic postconditioning is emerging as an effective method to protect injury due to ischemia and reperfusion in many organs, such as heart, lung, kidney, liver and intestine [24]–[30]. It is defined as a series of rapid intermittent interruptions of blood flow in the early phase of reperfusion that mechanically alters the hydrodynamics of blood flow [31]. In the clinical study, it was found that ischemic postconditioning could decrease heart edema caused by revascularization of stenotic artery [32] and protect endothelial function [29]. In the rat experiment, ischemic postconditioning could decrease brain edema and attenuate the disruption of BBB by inhibiting expression of MMP-9 and attenuating loss of the extracellular matrix proteins [33]. These results indicate that ischemic postconditioning might be effective in preventing vasogenic edema due to cerebral ischemia and reperfusion. Particularly, ischemic postconditioning could be performed before re-establishment of blood supply to brain, which makes it become a feasible method that could be used potentially in future clinical practice. Moreover, no reports proved that ischemic postconditioning had any negative effects by now. Despite we have demonstrated in previous study that ischemic postconditioning could rescue neuronal death following early relief of carotid stenosis [34], the effects of ischemic postconditioning on brain edema and the permeability of BBB is still unclear, we thus investigated as well this issue in this study via using a rat model of cerebral hypoperfusion.

## Materials and Methods

### Animals

Adult male Wistar rats (weighing 280–300 g) supplied by Jilin University Experimental Animal Center were housed in plastic cages (2 rats per cage) with soft bedding and free access to food and water at controlled room temperature (22–25°C) under a 12:12 h day/night cycle, and they were kept for 7 d before the experiments. All animal procedures were approved by the ethical committee for animal experiments, Jilin University, Changchun, China. Efforts were made to minimize animal suffering and to keep the number of animals used at a minimum.

### Surgical procedure and postconditioning protocol

A method described previously [34] was used in this experiment to make severe carotid stenosis with a slight modification. Briefly, the rats were fasted overnight and freely accessed to water. Anesthesia was induced with 5% halothane and maintained with 2% halothane in oxygen/nitrous oxide (30%/70%) gas mixture. Through a midline cervical incision, the skin and muscles were bluntly dissected, and the bilateral common carotid artery was exposed and freed from its sheath. The bilateral common carotid artery was bound with a stainless microtube with a diameter of 0.45 mm and a length of 0.5 cm. Subsequently, the bilateral common carotid artery was ligated using a 4-0 suture line soaked in dexamethasone at the proximal portion that was 0.5 cm from the bifurcation of the internal and external carotid artery. During the surgical procedure, a rectal temperature was maintained between 36.5 and 37.5°C. Rats were given aspirin as an anticoagulant (30 mg/L) in their drinking water 3 days after surgery.

**Figure 1:**
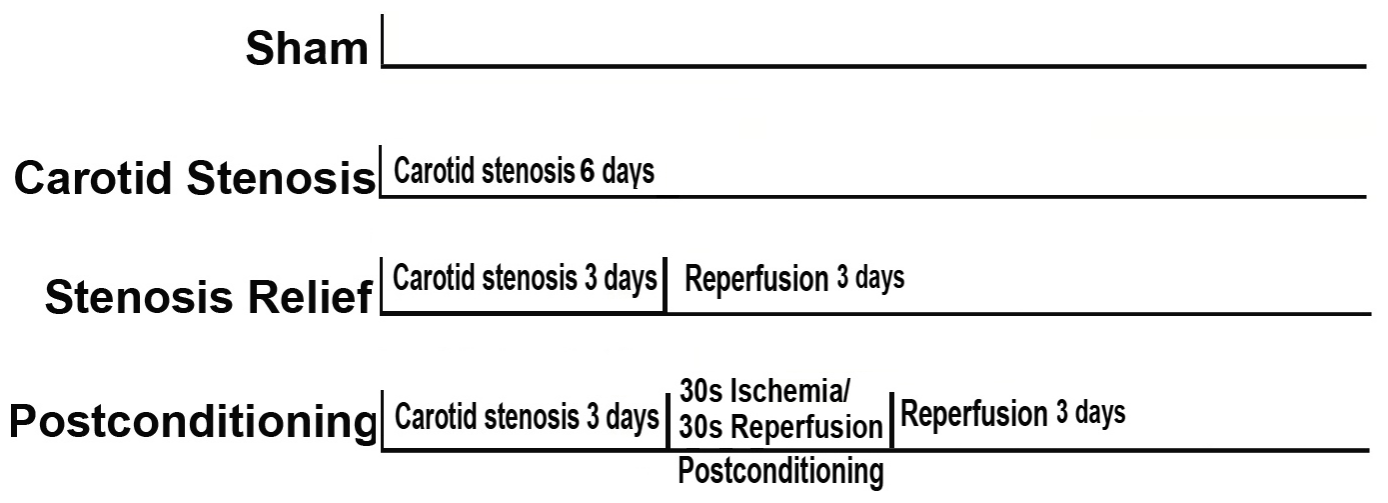
Schematic depicting the order of surgical procedures for Wistar rats undergoing sham operation, carotid stenosis, stenosis relief and ischemic postconditioning (ischemic postconditioning consisted of 3 cycles of 30-s/30-s reperfusion/ clamping after 3 days ischemic episode).

At the start of the study, the rats were assigned randomly into sham-operated group, carotid stenosis group, and ischemic postconditioning group according to a computer generated randomization schedule. In the sham-operated group, the bilateral common carotid artery was exposed, but no ligature was made. In the carotid stenosis group, the carotid stenosis was maintained 3 days before it was relieved. In the ischemic postconditioning group, three cycles of 30-s/30-s reperfusion/clamping were exerted at the end of reliving carotid stenosis (Fig. 1).

### Evaluation of brain edema

Brain edema was evaluated as describe previously [50]. Briefly, the rats from each group were deeply anesthetized and decapitated at day 1, day 2 and day 3 following relief of carotid stenosis. The brains were removed and weighted immediately to obtain the wet weight. Then, these brain tissues were dried in an oven at 120 °C for 24 h and then reweighed to obtain the dry weight. Cerebral water content was calculated according to the following formula: [(wet weight - dry weight)] / wet weight × 100%:

### Measurement of Blood-Brain Barrier permeability

Blood-Brain Barrier (BBB) permeability was assessed by the concentration of Evans' Blue (EB) and fluorescein sodium (NaF) in brain as described by Lenzsér et al [39]. The rats were anesthetized, administered intravenously EB and NaF (2% solution, 4 ml/kg) for 60 min, and perfused with 0.9% NaCl (1000 ml/kg). The brains were removed, and the cortex was isolated on ice and frozen in liquid nitrogen and stored in −80°C till to test. The samples were homogenized with 1 volume of PBS and then added 10 volumes of 50% trichloroacetic acid in to precipitate protein. The homogenate was centrifuged at 10,000 g for 20 min at 4 °C. For the measurement of EB, 100 µl supernatant was detected at 620/680 nm (excitation/emission wavelength) by microplate spectrophotometer (Bio-Rad, Richmond, CA, USA). Another homogenate was added NaOH to adjust pH value and detected in 485/535 nm (excitation/emission wave length). Calculations were based on external standards in the same solvent. The content of the dye was quantified by linear standard curves of EB (0.0763–1.2207 µg/ml) and NaF (0.0191–0.1526 µg/ml), and expressed by per gram tissue.

### SDS- PAGE Gelatin Zymography

SDS- PAGE gelatin zymography was performed as described previously[19]. In brief, the rats were deeply anesthetized and transcardially perfused with ice-cold PBS (pH 7.4). After the Brains were taken out, the cortex was isolated on ice, frozen immediately in liquid nitrogen and stored at −80°C. The cortex tissue were then homogenized with a Dounce homogenizer on ice in 10 volumes of lysis buffer containing 50 mmol/L Tris-HCI (pH 7.4), 150 mmol/L NaCl, 1%Nonidet P-40, 0.1% SDS, 0.1% deoxycholic acid, 2 µg/mL leupeptin, 2 µg/mL aprotinin, 1 mmol/L PMSF. The homogenate was centrifuged at 10,000 g for 5 minutes at 4°C. The supernatant was collected, and its total protein concentration was determined by using Bradford assay (Bio-Rad Laboratories, Hercules, CA, USA). Equal amounts of prepared protein samples (20 µg) were loaded and separated by 10% Tris-glycine gel with 0.1% gelatin as substrate. After electrophoretic separation, the gel was renaturated and incubated with developing buffer at 37°C for 24 h. Then, the gel was stained with 0.5% Coomassie Blue R-250 for 30 min and destained appropriately. Proteolytic bands in the zymography were quantified by scanning densitometry (Quantity one, Bio-Rad).

### In Situ Zymography

In situ zymography was performed as described previously[51]. The rats were deeply anesthetized and transcardially perfused with ice-cold PBS (pH 7.4). The brains were removed quickly and frozen on dry ice followed by in liquid nitrogen. Sections with thickness of 20 µm were cut on a cryostat and incubated at room temperature overnight in 0.05 mol/L Tris-HCl, 0.15 mol/L NaCl, 5 mmol/L CaCl2, and 0.2 mmol/L NaN3, pH 7.6, containing 40 µg of FITC-labeled gelatin (Molecular Probes, Eugene, OR). Gelatin with a fluorescent tagremains caged (no fluorescence) until it is cleaved by gelatinase activity. The cortex region used in this study was located at the dorsal lateral region to hippocampus. The in situ gelatinolysis was revealed by the appearance of fluorescent brain constituents. Reaction products were visualized by fluorescence microscope (Olympus Microsystems).

### Immunohistochemistry

Immunohistochemical staining was used to test the distribution and expression of **claudin-5 and occludin** in the microvessels of brain cortex and was performed as described by Jiao et al [52]. The rats were deeply anesthetized and transcardially perfused with ice-cold PBS (pH 7.4) for 3 min, followed by ice-cold PBS with 4% paraformaldehyde for another 3 min. Then, the brain tissue was removed and fixed 24 h in PBS solution containing 4% paraformaldehyde at 4 °C. Subsequently, brains were immersed in 30% sucrose solution with PBS for another 24 h. Coronal sections at the level of the anterior commissure were cut into pieces 10 µm thick. Sections were incubated overnight with rabbit polyclonal anti-claudin-5 antibody (diluted 1∶150; Santa Cruz Biotechnology, Santa Cruz, CA, USA) and rabbit polyclonal anti-occludin anti-body (diluted 1∶150; Santa Cruz Biotechnology). The remaining procedures conformed to the standard procedures.

### ELISA assay

The rats were deeply anesthetized and transcardially perfused with ice-cold PBS (pH 7.4). After the Brains were taken out, the cortex was isolated on ice, frozen immediately in liquid nitrogen and stored at −80°C. The cortex tissues homogenized on ice with a Dounce homogenizer by using a lysis buffter containing 10 mmol/L HEPES (pH 7.9), 10 mmol/L KCL, 0.1 mmol/L EDTA, 0.1 mmol/L EGTA, 1 mmol/L DTT, 1.0 mmol/L PMSF, 2 µg/mL leupeptin, 2 µg/mL aprotinin, and 10 mg/mL phosphokinase inhibitor. The homogenates were centrifuged at 10,000 g for 30 min at 4°C, and the supernatant was collected. Then, its total protein concentration was determined by using Bradford assay (Bio-Rad Laboratories, Hercules, CA, USA).The samples were added in quadruplicate in wells (0.1 µg/well), and incubated overnight at 4 °C. After washing with PBS, coated wells were blocked 1 h in PBS containing 1% BSA and 0.05% Tween-20 and then incubated with rabbit anti-MMP9 polyclonal antibody (diluted 1∶1000; Santa Cruz Biotechnology, Santa Cruz, CA, USA), polyclonal rabbit anti-claudin-5 (diluted 1∶500; Santa Cruz Biotechnology), rabbit anti-occludin (diluted 1∶500; Santa Cruz Biotechnology), or rabbit anti-actin polyclonal anti-body (1∶2000; Santa Cruz Biotechnology Inc.) overnight at 4 °C. Antibody binding was visualized using a horseradish peroxidase-conjugated goat anti-rabbit secondary antibody (diluted 1∶2000; Santa Cruz Biotechnology Inc.). Color was developed by tetra-methylbenzidine (Sigma) as a substrate, and reaction was stopped by addition of 50 µL of 2 mol/L H2SO4. Optical density (OD) was measured at 450 nm using a microplate reader (Bio-Rad, Richmond, CA, USA). The ratio of the target protein OD to the actin OD was used to represent the alteration in protein level.

### Data analysis

All data are expressed as Mean ± SD and analyzed statistically by SPSS 17.0 software (SPSS Corp, Armonk, NY, USA). One way ANOVA was used as appropriate for comparison between different groups. Each test was repeated at least four times and each group consisted of four rats. *P*<0.05 was considered statistically significant.

## Results

### Ischemic postconditioning suppressed brain edema caused by stenosis relief

**Figure 2:**
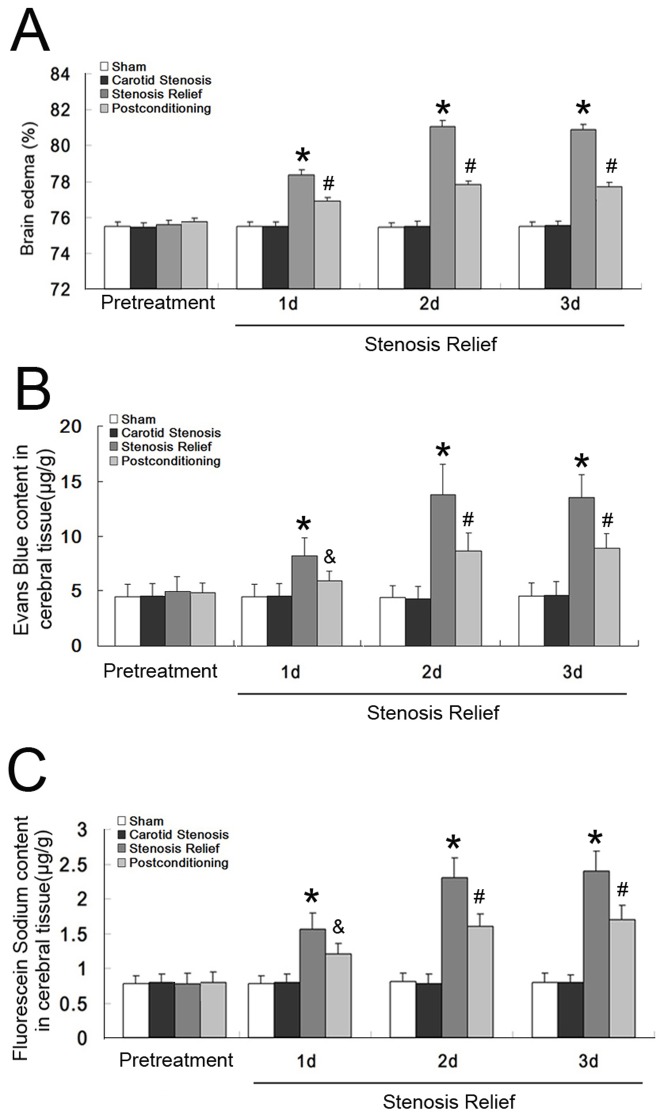
Measurement of brain water content and cerebral concentration of EB and NaF. A, Brain water content. Following relief of carotid stenosis, brain water content increased significantly when compared with that in the carotid stenosis group at each indicated time point. However, the elevated brain water content was mitigated markedly by ischemic postconditioning. B, Cerebral concentration of Evans' Blue (EB); C, Cerebral concentration of fluorescein sodium (NaF). Relief of carotid stenosis made the cerebral level of EB and NaF increase significantly at each corresponding time point, which was inhibited by ischemic postconditioning performed administrated prior to stenosis relief. This indicated that, ischemic postconditioning is an effective method to suppress brain edema and elevated BBB permeability caused by relief of carotid stenosis. *: *p*<0.01, versus carotid stenosis group; #: *p*<0.01, versus stenosis relief group; &: *p*<0.05, versus stenosis relief group.

As shown in figure 2A, brain water content remained stable at the level of about 75.48% ±0.26% in the carotid stenosis group, and no significant difference was found at any corresponding time point between the carotid stenosis group and the sham group. By contrast, when carotid stenosis was relieved, brain water content increased respectively to 78.37%±0.28% at day 1, 81.21%±0.34% at day 2 and 80.78%±0.29% at day 3, which were significantly higher than those in the carotid stenosis group at each corresponding time point. However, the elevated brain water content caused by stenosis relief was mitigated significantly by administration of ischemic postconditioning to 76.89%±0.19%, 77.84%±0.21%, and 76.97%±0.22% from day 1 to day 3. This result indicated that, ischemic postconditioning is an effective method to suppress brain edema caused by relief of carotid stenosis.

### Ischemic postconditioning inhibited the elevation of BBB permeability caused by stenosis relief

Disruption of BBB is thought to be one of the factors leading to cerebral edema, we thus examined the permeability of BBB by measuring cerebral content of EB (Evans' Blue) and NaF (fluorescein sodium). As figure 2 B and C showed, no significant difference was found in the brain content of EB and NaF between the carotid stenosis group and the sham group. However, following relief of carotid stenosis, the cerebral level of EB and NaF became significantly higher than those in the carotid stenosis group at each corresponding time point. The level of EB increased to 8.23±2.18 µg/g at day 1, 13.8±2.71 µg/g at day 2 and 13.51±2.26 µg/g at day 3, and NaF level rose to 1.72±0.23 µg/g, 2.33±0.29 µg/g and 2.39±0.31 µg/g, respectively. By contrast, after application of ischemic postconditioning, the levels of EB were 5.93±0.95 µg/g, 8.68±1.58 µg/g and 8.92±1.33 µg/g, and NaF were 1.22±0.15 µg/g, 1.65±0.18 µg/g and 1.73±0.21 µg/g from day 1 to day 3, both of which were lower markedly than those in the stenosis relief group at each corresponding time point. These results indicated that ischemic postconditioning could inhibit BBB disruption caused by relief of carotid stenosis.

### Ischemic postconditioning attenuated the activity and expression of MMP-9

**Figure 3:**
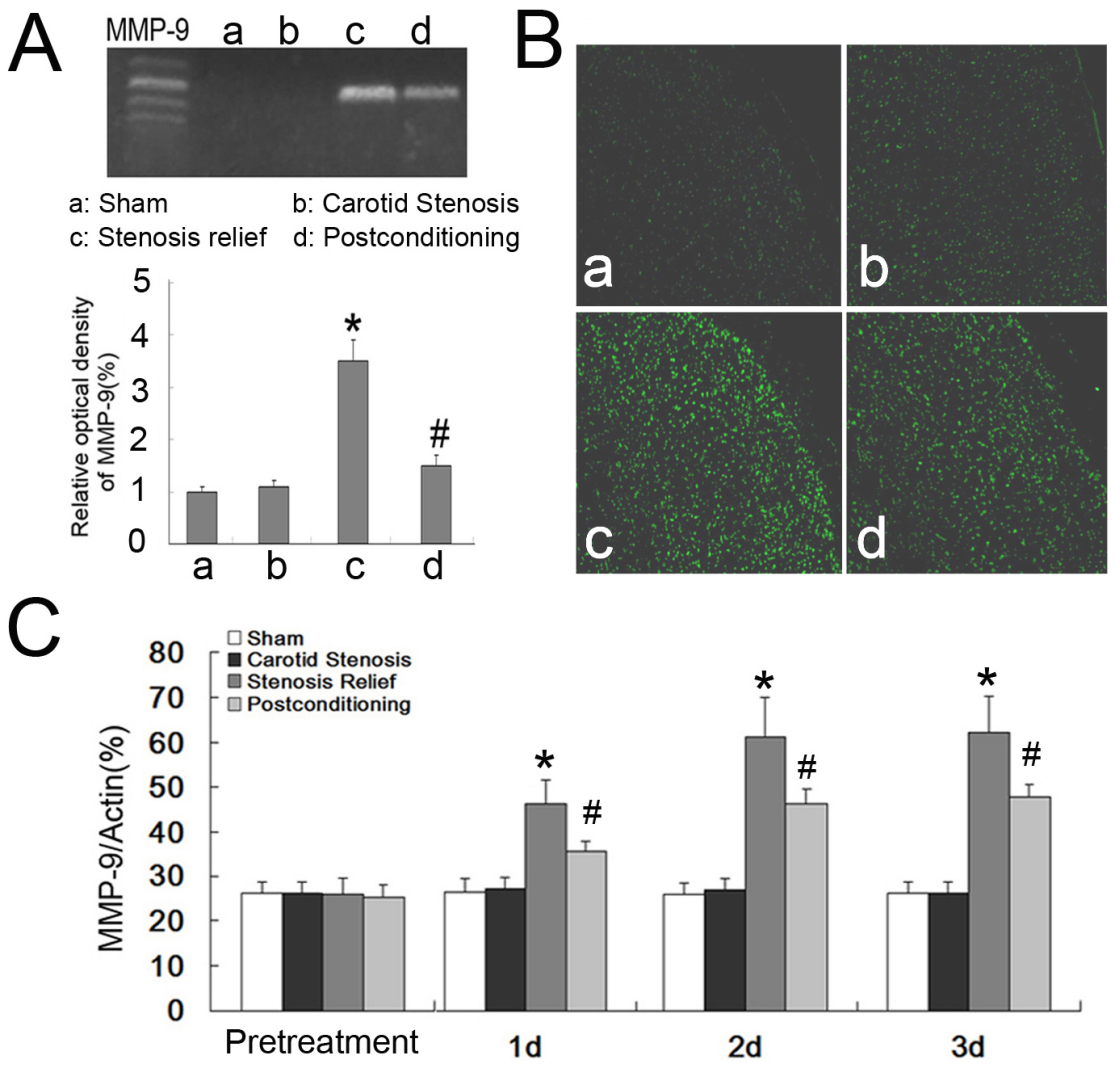
Measurment of gelatinase activity and expression of MMP-9. A, gel gelatinase activity of MMP-9 and statistical analysis. The gelatinase activity of MMP-9 in the stenosis relief group was 3.52±0.43 times higher than that in the carotid stenosis group at day 2 following relief of carotid stenosis, but it was alleviated significantly by ischemic postconditioning. B, in situ gelatinage activity of MMP-9. Relief of carotid stenosis made the FITC signal representing gelatinase activity become stronger when compared with that in the sham and carotid group. However, the increased signal intensity was inhibited markedly by ischemic postconditioning. C, ECL assay of MMP-9. The expressional levels of MMP-9 in the stenosis relief group were significantly higher than those in the sham and the carotid stenosis group. By contrast, this elevation was mitigated from day 1 to day 3 after application of ischemic postconditioning. *: *p*<0.01, versus carotid stenosis group; #: *p*<0.01, versus stenosis relief group.

Previous studies have shown that MMP-9 played an active role in modulating the permeability of BBB [17]–[19], we thus speculated that stenosis relief might cause aberrant activity and expression in MMP-9. Because brain edema and the permeability of BBB reached peak value at day 2 when carotid stenosis was relieved (Figure 2), we chose this time point to assay MMP-9 activity in the rats treated with ischemic postconditioning or stenosis relief alone. As shown in figure 3 A, gelatin zymography revealed that the gelatinase activity of MMP-9 in the stenosis relief group was 3.52±0.43 times higher than those in the carotid stenosis group (*P*<0.01), but it was alleviated to 1.48±0.37 times by ischemic postconditioning. Similarly, in situ zymography showed that the FITC signal which represents gelatinase activity was weak in the sham and carotid stenosis group (Figure 3 B), but increased markedly in the stenosis relief group and was suppressed by ischemic postconditioning. To elucidate the factors affecting the increased activity of MMP-9, we analyzed its expressional level by ELISA. As figure 3 C showed, the expressional levels of MMP-9 in the stenosis relief group increased to 46.33±5.31, 61.2±8.65, and 62.1±8.11 at day 1, day 2 and day 3, respectively (*P*<0.01 versus carotid stenosis group at each scheduled time point). However, the elevation was mitigated from day 1 to day 3 after application of ischemic postconditioning was inhibited to 35.6±2.21, 46.2±3.18, 47.5±2.87 (*P*<0.01 versus stenosis relief group at each time point). These data indicated that expressional up-regulation underlies the increased activity of MMP-9 due to reestablishment of cerebral blood supply.

### Ischemic postconditioning suppressed quantitative reduction of claudin-5 and occludin caused by stenosis relief

**Figure 4:**
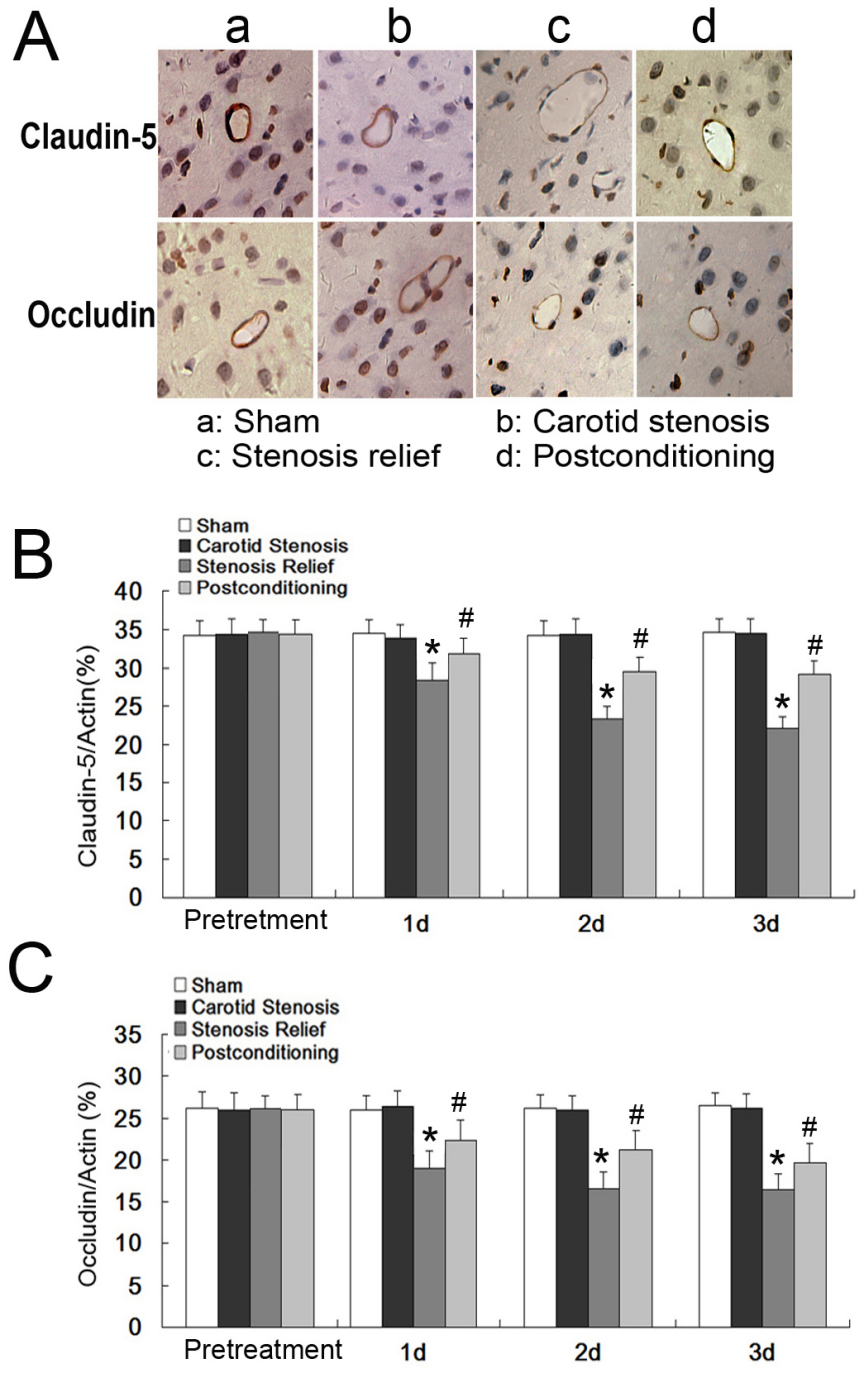
Distribution and expression of tight junction proteins caludin-5 and occludin. A, distribution of caludin-5 and occludin in cerebral microvessels. Immuno-histochemical images showed that claudin-5 and occludin were located sharply and continuously on cerebral microvessels in the sham and carotid stenosis group. At 2 day following relief of carotid stenosis, their distribution became discontinuous, which was reversed partly by ischemic postconditioning. B, ELISA assay of claudin-5 concentration. C, ELISA assay of occludin concentration. The level of caludin-5 and occludin in stenosis relief group were significantly lower that those in the sham and the carotid stenosis group at each scheduled time point. However, ischemic postconditioning maintained their quantity. *: *p*<0.01, versus carotid stenosis group; #: *p*<0.01, versus stenosis relief group.

In this section, we tested the distribution of tight junction proteins claudin-5 and occludin on cerebral microvessels and analyzed their changes in quantity. Immuno-histochemical images showed that claudin-5 and occludin were both located sharply and continuously on cerebral microvessels in the carotid stenosis group (Figure 4 A), but they became discontinuous (not circling the cerebral microvessels) in stenosis relief group. However, ischemic postconditioning reversed partly this alteration caused by stenosis relief. For quantifying the quantitative changes of claudin-5 and occludin, ELISA assay was performed. As shown in figure 4 B and C, in comparison with those in the sham and the carotid stenosis group, the level of claudin-5 and occludin decreased significantly from day 1 to day 3 in the stenosis relief group. Nevertheless, ischemic postconditioning suppressed the reduction in these two proteins at each corresponding time point (*P*<0.01, versus carotid stenosis group). Considering claudin-5 and occludin were the constituents of BBB, we thought that the protection of ischemic postconditioning on permeability of BBB is via maintaining the quantity of claudin-5 and occludin in BBB.

## Discussion

In this study, we found that development of brain edema and elevation of permeability of BBB following early relief of carotid stenosis were associated with increased activity and expression of MMP-9 and decreased quantity of tight junction proteins claudin-5 and occludin. However, this alteration was suppressed by ischemic postconditioning. The method used in this study has been demonstrated to be effective in establishing rat model of carotid stenosis and cerebral hypoperfusion. The extent of stenosis was dependent on the diameter of the microtube bound to carotid artery. Angiography showed that a microtube with a diameter of 0.45 mm could lead to severe carotid stenosis (the inner diameter of carotid artery<70%) in adult rats [34]. Thus, as described previously, we used the microtubes to produce severe carotid stenosis and hypoperfusion in rats to imitate similar conditions in human being.

It is well known that BBB plays an important role in protecting the neuronal microenvironment via restricting the movement of molecules from cerebral capillary to brain tissue. The loss of BBB integrity would lead to cerebral hemorrhage, vasogenic edema and neuronal cell death [35]. In this study, we evaluated the permeability of BBB by examining the content of EB and NaF within brain, as well as examined the brain water content. We found that, at the same time that water content increased following relief of carotid stenosis, BBB permeability also elevated from day 1 to day 3. This indicated that brain edema caused by stenosis relief is associated with increased BBB permeability. By contrast, both brain edema and abnormal BBB permeability were suppressed by ischemic postconditioning administrated prior to stenosis relief. This is also consistent with prior clinical finding that ischemic postconditioning decreased heart edema caused by revascularization of stenotic artery [32]. In the previous study, we found that CBF increased from the decreased level 45% at day 3 after carotid stenosis was produced, to 78% at day 4 when carotid stenosis was relieved [34]. Despite it was reported that ischemic postconditioning effectively regulated the cerebral blood flow to the ischemic region via blocking hyperperfusion or hypoperfusion [36], our previous results showed that ischemic postconditioning did not change cerebral blood flow significantly when compared with that in the group treated by relief of stenosis alone. Thus, we think that the suppression of ischemic postconditioning to brain edema and increased BBB permeability is not via modulating cerebral blood flow.

At the interface between blood and brain, endothelial cells and associated astrocytic foot processes form “tight junctions” [23]. Tight junction proteins occludin and claudin-5 forms the endothelial barrier, and is now well accepted as a major determinant for paracellular permeability [37]. It was found that disruption of the TJ barrier directly involved in the pathogenesis and aggravation of cerebral ischemia - reperfusion injury. In this study, we proved that claudin-5 and occludin decreased following relief of carotid stenosis. Moreover, immunohistochemistry revealed that TJ between the microvascular endothelial cells in the ischemic brain exhibited improved integrity as visualized by the localization of claudin-5 and occludin after treatment of ischemic postconditioning. However, ischemic postconditioning maintained their quantity, indicating ischemic postconditioning preserves the BBB intact via re-establishing the tight junction barrier. Besides the tight junction proteins, it was found in the clinical study that ischemic postconditioning protected endothelial function. Human study showed that damaged brachial artery endothelial function by ischemia and reperfusion was protected by ischemic postconditioning (consisting of 3 cycles 30 seconds ischemia and 30 seconds reperfusion) [28], [29]. Moreover, Yan J reported PUMA (p53 upregulated modulator of apoptosis) induction of endothelial cell apoptosis from the endoplasmic reticulum facilitated BBB disruption following subarchnoid hemorrhage[38].

Previous study showed that clear increase of BBB permeability was detected at the same time as the appearance of activated MMP-9 [39]. Other researchers proved as well that MMP-9 affected the permeability of BBB [40], [41]. It is thought that the impact of MMP-9 on BBB permeability is via modulating the tight junction proteins and basal lamina proteins, which is supported by the evidence that up-regulated expression of MMP-9 was accompanied by reduction in basal lamina proteins laminin and fibronectin [33]. Thus, we compared gelatinase activity and expression of MMP-9 in each group. We found that both the gelatinase activity and the expression of MMP-9 increased significantly in the stenosis relief group, but they were inhibited by administration of ischemic postconditioning. This finding was consistent with previous report that ischemic postconditioning could diminish the expressional elevation of MMP-9 caused by acute focal ischemia and reperfusion [33]. Additionally, we found as well that elevation in the activity and expression of MMP-9 was in coincidence with reduction in claudin-5 and occludin, suggesting the regulation of MMP-9 to BBB permeability might be via degradation of tight junction proteins claudin-5 and occludin. The protective effects of ischemic postconditioning on organ damage caused by ischemia and reperfusion have been investigated widely. Thus, the protective mechanism of ischemic postconditioning is thought to be associated with inducing endogenous protective effects, including inhibition of endoplasmic stress, modulation of signal pathway, suppression of oxidative stress, regulation of enzyme activity, and receptor activation [42]–[47]. Considering that matrix metalloproteinases is a family of enzymes that are activated by oxidative stress [48] and ischemic postconditioning could inhibit oxidative stress caused by relief of carotid stenosis [34], we speculate that the inhibitory effects of ischemic postconditioning on MMP-9 is due to its inhibition of oxidative stress, which is also supported by the evidence that administration of free radical scavenger edaravone inhibited breakdown of BBB [49]. However, it is still needed to be clarified whether or not other mechanism involve in the protection of ischemic postconditioning.

## Conclusion

Our study showed that early relief of severe bilateral carotid stenosis could lead to brain edema and elevation of BBB permeability, which was associated closely with increased activity and expression in MMP-9 and decreased quantity in tight junction proteins claudin-5 and occludin. By contrast, administration of ischemic postconditioning prior to relief of carotid stenosis suppressed the elevation in MMP-9 activity and expression and maintained the level of claudin-5 and occludin. Therefore, our study indicates that ischemic postconditioning is an effective method that could be used to prevent brain edema and BBB disruption caused by early relief of heavy carotid stenosis.
